# Patterns of sexual and HIV-related stigma among men who have sex with men and women living with HIV in Haiti

**DOI:** 10.1038/s41598-022-11647-1

**Published:** 2022-05-07

**Authors:** Jessy G. Dévieux, John Vertovec, Michèle Jean-Gilles, Rhonda Rosenberg, Cassandra René, Elena Cyrus, Samuel Emieux Jean, Willy Dunbar

**Affiliations:** 1 Department of Health Promotion and Disease Prevention, Robert Stempel College of Public Health & Social Work, Florida International University, Biscayne Bay Campus-Academic 1, 277A, 3000, NE 151st Street, Miami, FL 33181 USA; 2 Behavioral Science Research Institute, Miami, FL USA; 3 REACH-Research Network for Health and Society, Kimberly Green Latin American and Caribbean Center, Florida International University, Miami, FL USA; 4 College of Medicine, University of Central Florida, Orlando, FL USA; 5 Population Services International/Organisation Haïtienne de Marketing Social Pour la Santé, Port-au-Prince, Haïti

**Keywords:** Public health, Infectious diseases

## Abstract

Vulnerability to contracting HIV among Men who have Sex with Men and Women (MSMW) was recognized early in the epidemic. However, while global HIV efforts have made tremendous progress for the heterosexually-identified population, the specific needs of MSMW were not directly addressed with tailored and context-adapted interventions. The purpose of this study was to inform this area of research by exploring patterns of stigma through sexual identity developmental history as well as coping mechanisms among MSMW living with HIV in Haiti. A qualitative descriptive study comprised of in-depth interviews with 32 MSMW living with HIV was carried out. Participants were recruited using snowball techniques. An inductive thematic analysis was conducted in NVivo, contextualized by the socio-ecological context of Haiti. MSMW reported struggling with their sexuality since their adolescence, often because of enacted stigma from family members, the community, and cultural conflicts. Most participants described experiencing anxiety, psychological distress, depression, social isolation, suicidal ideation and suicide attempts. Mechanisms for coping with stigma included self-acceptance, social support, hiding their sexual orientation, and tolerance of the voodoo religion. To combat stigma, and improve HIV treatment adherence and retention among MSMW, culturally-tailored multilevel initiatives should be implemented.

## Introduction

Haiti has the greatest number of cases of HIV in the Caribbean and the prevalence is among the highest in the region^1,2^. For the past three decades, national HIV strategic responses have been instrumental in reducing Haiti’s HIV prevalence from 6.2% in 1993 to 1.9% in 2020^3,4^. As the country continues to make progress towards controlling the epidemic and expanding access to antiretroviral therapy (ART), Men who have Sex with Men and Women (MSMW), are largely left out of the discussion of key populations^5–7^. Yet according to a survey conducted and published by the United Nations, the HIV prevalence among MSMW was 18.2% in 2017, making them the most underserved population in terms of HIV prevention and care^4^.

Since the beginning of the epidemic, studies have shown that MSMW are disproportionately impacted by HIV and, when infected, represent a bridge for the spread to the general population of heterosexuals^8,9^. With the availability of ART, adherence and viral suppression are critical to improving their health and preventing new infections^10,11^. However, retention in HIV services is poor due to various barriers that continue to prevent MSMW from accessing care and being retained in treatment as a means of achieving the full benefits of ART^12^. Among these barriers, sexual and HIV-related stigma and discrimination remain major concerns and underlie social vulnerability^13^. As MSMW are highly stigmatized by both religious and social norms, homosexual practices are often driven underground. Perpetuated at the community level, violence toward MSMW is sometimes overlooked by the police force^7^. Moreover, access to HIV prevention and treatment services remain limited due to fear of non-voluntary disclosure and the fact that healthcare providers are perceived as judgmental and unable to respect confidentiality^5,12^.

The seminal works of Erving Goffman demonstrate how people may be affected by three types of stigmatized conditions: physical deformity, character blemishes, and prejudice^14^. The general population perceives character blemishes among individuals with HIV and who are homosexual^14^. Stigma, based on sexual orientation, is rationalized and justified by the ideological systems of society for any non-heterosexual behavior, identity, relationship, or community^15^. Research has shown that sexual stigma is associated with greater HIV risk behaviors among MSMW who have unprotected anal intercourse with men, as well as concurrent unprotected sex with both men and women^16^. Furthermore, for those living with HIV, they may be stigmatized because of preexisting marginalized behaviors and identities coupled with misconceptions about the disease and its transmission, fears of contagion, and negative portrayal^17^. Thus, these forms of stigma are interlinked and are important to assess because they often impact behavior and health outcomes^18^. Evidence indicates that stigma can be considered a fundamental barrier for prevention and treatment of HIV in at-risk populations, including MSMW^19,20^. Based on their sexuality, behavior, and their HIV status, MSMW confront multiple layers of ostracism and discrimination which hamper their linkage and retention into care and continue to pose a real threat to control of the epidemic^21^.

Despite the documented impact of stigma on HIV risk among MSMW, and its role in the exacerbation of HIV risk and transmission for other individuals in their networks and communities, limited work has been done to address vulnerabilities particular to MSMW that are crucial to increasing their access to prevention and treatment tools, and to reducing HIV infection within their sexual networks. There remains relatively limited tailored interventions that consider the context, developmental history, patterns of stigma, relations to mental health and the behavioral mechanisms in resource limited settings like Haiti due to lack of data^22^.

To address this gap and provide an improved understanding of the context of stigma, we conducted a qualitative study in Haiti to: (1) explore the patterns of sexual and HIV-related stigma through MSMW’s developmental history, (2) assess the impact of stigma on their mental health, and (3) describe different levels of stigma and coping mechanisms used.

## Methods

### Qualitative study design

We used a qualitative descriptive design by employing in-depth interview data collection techniques based on grounded theory and inductive approaches for thematic content analysis^23,24^.

### Recruitment of participants

Using snowball sampling procedures, which involved systematic diversification, a sample of 32 individuals were recruited to participate in the study^25^. Eligible participants, age ≥ 18 years, were men who reported sexual behavior with both men and women, were fluent in Haitian Creole, and living with HIV. Participants were recruited from community-based organizations that provide services to the LGBTQ community in Port-au-Prince, the capital of Haiti and were approached by trained peer research assistants who explained the purpose of the study. Those who agreed to be part of the study provided informed consent, were assigned a participant number, and received an incentive payment upon completion of a one-time in-depth individual interview. All interviews were conducted in Haitian Creole and lasted between 60 and 90 min. Although snowball through referral was the recruitment process, systematic diversification was achieved through inclusion of participants with different demographic characteristics in terms of age, education level, income, employment status and living arrangements. Participants were between 20 and 42 years of age, with a median age of 31. Most of them had attended secondary school, were living with sex partners, working with a monthly income between $ 50 and $ 200 USD (Table 1).

**Table 1:** Demographic characteristics of 32 participants.

Characteristic	Category	Participants n (%)
Age (years)	20–25	7 (21.9%)
	26–30	8 (25.0%)
	31–35	9 (28.1%)
	36–42	8 (25.0%)
Education	Primary	6 (18.8%)
	Secondary	21 (65.6%)
	Higher education	5 (15.6%)
Living with	Roommate	5 (15.6%)
	Sex partner	24 (75.0%)
	Wife	3 (9.4%)
Employment	Employed	19 (59.4%)
	Unemployed	10 (31.2%)
	Students	2 (6.2%)
Monthly income (USD)	Less than $ 50	13 (40.6%)
	$ 50–$ 200	14 (43.8%)
	$ 200–$ 500	4 (12.5%)
	Missing	1 (3.1%)

### Data collection

In-depth interviews were conducted by trained research assistants. All interviews took place in private rooms at clinical settings and were conducted in Haitian Creole. Participants were advised they could decline to answer questions or stop the interview at any time. The interviews followed a topic guide that included personal and demographic background, sexual attraction and preferences throughout their developmental history, childhood and adolescence experiences of violence and trauma, history of depression, alcohol and other drug use, sexual experiences, condom-use practices, serostatus of sexual partners, disclosure of HIV status to sexual partners, stigma coping strategies, and the role of religion. The guide was adapted during fieldwork to accommodate emerging themes. Participants were free to ask questions on issues they felt were not clear and permission was obtained to audio record the interviews.

### Coding and data analysis

Audio-taped recordings were transcribed verbatim in Haitian Creole, then translated into English by two research assistants fluent in both Haitian Creole and English. Two FIU faculty researchers, fluent in Haitian Creole and English, reviewed all translated transcriptions to conduct quality assurance checks.

All the transcriptions were uploaded into an NVivo Qualitative Data Analysis Software (version 12.1) database. Codes were then developed inductively to establish novel themes from the interviews themselves. Grounded theory guided this process; that is, theoretical perspectives (and their associated codes) were generated, debated, and discussed as we moved through the database. This approach offered the advantage of organizing the analytical process into a series of sequential steps which allowed us to discover patterns of stigma and coping mechanisms^26^.

First, a group of six coders collectively read through three transcripts to inductively identify broad as well as specific codes relevant to the study objectives. The goal for this initial round of coding was to create the study’s code book in alignment with the broader research questions guiding the study’s interview procedures. After the initial round, 11 broad codes that were relevant to the main concepts of the transcribed data, were identified and defined. In reviewing the data, we further developed sub-codes that presented specific relationships that emerged from the transcriptions. As coders reviewed and analyzed the interview data, memos were also written within NVivo to note any thoughts related to the study’s objective. A second round of coding was conducted to further refine the codes and code descriptions in the code book.

After completing the two rounds of coding, the six coders were paired and assigned transcripts to individually code based on the finalized code book. Upon completion of the coding, the pairs scheduled meetings to discuss the assigned codes and memos throughout the transcripts. Once the pairs reached consensus on the codes within each transcript, a final document was uploaded into NVivo. Reliability was assessed by ensuring 85% coding agreement as a benchmark measure of consensus. Interview quotations that best described the themes were selected for presentation, while also aiming to incorporate examples from most participants and all age groups.

As with any research team, power asymmetries between more ‘tenured’ researchers and research assistants (i.e., some of the coders) was always a possibility. Using sequential rounds of coding and codebook development helped mitigate power imbalances and the use of memos was a thoughtful strategy that encouraged all the coders, regardless of position within the academy, to contribute their thoughts with more time to think through them than if they were forced to contribute them “on-the-spot” in an in-person meeting. Furthermore, all the coders were encouraged to come up with codes and analyses that were in line with their own research interests or personal lived experiences, thereby giving them even more space to contribute to the data analysis process.

To support the trustworthiness of the research, we used credibility, dependability, conformability and transferability^27,28^. Prior to data collection and in order to understand the contextual factors relating to the life and care for MSMW living with HIV, meetings were held with the stakeholders. To ensure credibility, in-depth interviews were conducted by peer trained research assistants who were familiar with the context of the target population. Dependability was established by describing the data analysis in detail and providing direct citations to reveal the basis from which the analysis was conducted. In order to maintain accuracy, the citations used were translated from Haitian Creole into English by experienced translators and researchers. The conformability and consistency of the analysis were established by discussing preliminary findings, emerging codes and themes until consensus was reached. To enhance the transferability of the findings, a description of the context, selection and demographics of participants, data collection and process of analysis is provided to enable the reader to determine whether the results of this study are transferable to another context.

### Ethics approvals

The study protocol was approved by the Haiti National Ethics Committee and the Florida International University (FIU) Institutional Review Board (IRB).

### Consent to participate

All participants gave written and oral informed consent.

### Consent for publication

The authors consent to publish.

## Results

While our analysis allowed for emergent findings within the study domains, our analytic approach was designed to examine how the factors found in the narratives interact in a systematic sample of MSMW living with HIV in Haiti, thereby contributing to the achievement of the aim of the study.

We first report the experiences of stigma stemming from heteronormative construction of gender roles and the experiences of sexual and HIV-related stigma during childhood and adolescence and current living conditions. Furthermore, we present themes related to the consequences for mental health. Finally, we describe stigma coping mechanisms.

### Stigma: experiences through sexual identity developmental history and current living conditions

Participants were asked to describe their experiences while growing up, their sexual development, with a focus on gender attraction and preferences, as well as factors determining stigma and discrimination. Their responses overwhelmingly indicated that sexual conflicts during their developmental phase were mainly based on family education on heteronormativity and representation of gender and gender roles. When asked to reflect on the stigma they had faced regarding their sexual orientation during their childhood, experiences of enacted stigma typically came in the forms of family violence, emotional abuse, verbal harassment, and employment discrimination.

Analysis of the in-depth interviews identified five main themes for experiences of stigma through developmental history and current living conditions. (1) ‘Social construction of heteronormativity’ encompasses childhood experiences associated with gender roles. Later on, homosexual orientation leads to rejection, family violence and emotional abuse. (2) ‘Verbal and physical harassment in the community’ and (3) ‘employment discrimination’ comprise the frequent rejecting reactions participants faced in the community due to their sexual orientation, which facilitated concealment of sexual identity. (4) ‘Christianity religious influences’ reinforce stigma, even from close family members. In contrast, the spiritual tenets of voodoo incorporate acceptance of all people of all sexual orientations and gender expressions. (5) ‘Sexual and HIV-related stigma’ resulted in a “double victim” effect and increasing barriers in the provision or uptake of efficacious HIV prevention and treatment services.

#### Social construction of heteronormativity

Heteronormativity refers to norms related to gender and sexuality that seek to reinforce existing power structures and ideologies, such as patriarchy and compulsory heterosexuality. According to Adrienne Rich, compulsory heterosexuality refers to the assumption of a male-dominated society that the only normal sexual relationship is between a man and a woman. The same observation is made in Haiti, influenced by Christian religion, where the culture of patriarchal society insists on compulsory heterosexuality^29^. In order to understand the sources and aspects of stigma, we asked participants to describe their developmental history. Their responses helped reveal the social construction of heteronormativity where men and women have their specific characteristics and tasks in the household and community:

“They told me that a man is a person who is supposed to find work to take care of himself and his family. He is the head of the house, and the woman she’s the one that makes food to give to the man and takes care of the house. She’s supposed to wear skirts. She’s supposed to have hair. It’s just the role society decided on how you are supposed to be.” IDIParticipant001.

The heteronormativity process elevates heterosexuality as natural and normal and devalues other sexual orientations, gender roles and relationship paradigms. Therefore, this situation reinforces rejection, ostracism and discrimination and contributes to homosexuality-related enacted stigma and violence. Experiences during childhood, within the broader socio-cultural environment, result in emotional neglect and lead to early and long-lasting impacts on mental health, drug use, and coping structures.

Furthermore, while some respondents reported strong relationships with family members, the majority described dysfunctional or superficial relationships with their parents, characterized by abandonment, arguments, and physical or verbal abuse:

“It’s friends who went to tell my mother that I was a homosexual, that I’m sick, and who put me in the problems with her, and she didn’t even ask me anything at all about it and she acted out. Because she spread the word in the area.” IDIParticipant002.

#### Verbal, physical harassment and employment discrimination

The experience of violence is known to be independently associated with risk behaviors for HIV infections^30^. When we asked questions regarding their current living conditions and experiences in the community, violence as a consequence of stigma including physical assault, was recalled by several participants, sometimes with immediate consequences on their rights to live freely in their country:

“Because the owner of the house said people better not be doing anything gay in their house. They asked me to leave the house. Because they will not live with a gay person inside their house. And that hurt me a lot, and it still hurts me a lot.” IDIParticipant003.

“I started suffering when they said that “masisi” (gay slur) don’t live here. Here is not for them and they vacate the premises. Then, I was hit with a bottle, my head was injured”. IDIParticipant004.

Although not commented on by everyone in the sample, enacted stigma was also experienced through employment discrimination, beyond the other forms of discrimination like housing discrimination described above. Indeed, several participants described their sexual orientation as a barrier to getting employed thus reinforcing their vulnerability and precarity, and which could lead to other HIV risk behaviors:

“I can remember a humiliation that I suffered looking for work because they told me they do not accept homosexuals in their job; I don’t know how they knew I was gay because I am not effeminate.” IDIParticipant006.

#### Christian religious influences

As the process of devaluation of MSMW occurs in the larger context of intersecting systems and hierarchical power dynamics, we wanted to explore the role of religion in the social structures that shape stigma manifestations. In a country that is predominantly Roman Catholic, homosexuality is largely condemned.

Within this framework, participants described how Christian religious communities foster moral judgments by promoting negative attitudes toward them, even from close family members:

“I was baptized and a practicing Baptist since I was 13 years old. I used to go to church up until I was 17 years old. But when the messages were given on sex, on the life of men, that are living with men, or for women living with women, that those things aren’t good. They talked about Sodom and Gomorrah so they could justify it (that being gay wasn't good). The Baptist church made it known, when a person is gay you shouldn’t ever tell them good morning. Everyone was enemies with me, [..because of my sexual orientation.] my aunts and my friends; they were not even saying good morning to me. The school teachers weren’t saying good morning to me.” IDIParticipant007.

#### Sexual and HIV-related stigma: “double victim” effect

Stigma and marginalization concurrently act as factors that amplify the risks for HIV. The prior syndemic factors interact to increase the vulnerability of MSMW and confer excess risk to HIV infection—what we refer to here as the “double victim effect”^31,32^. Findings indicated that MSMW are situated at the intersection of HIV‐related stigma and prejudice against their identities and behaviors, which exacerbate their experiences of stigma and discrimination.

“When they arrested me, that hurt me. And there is an event that marked me, it’s when I publicly declared that I was homosexual at a World AIDS day commemoration event. I got a lot of pressure and I had to leave the village for a while. And I recall even the people in my family, my uncle, my mother’s brother, were enemies with my grandma, who was his mother, because she tolerated me in her house.” IDIParticipant008.

### Stigma: impact on mental health

Existing studies of MSMW have demonstrated significant associations with mental health problems and social isolation. These data have documented that perceived stigma has deleterious consequences for mental health, including depression and anxiety^31^. Consequently, negative psychosocial factors are associated with increased HIV-risk behaviors and poorer HIV outcomes, including more frequent unprotected anal sex, and reduced engagement in HIV care and treatment^10,33^. Our findings identified significant psychosocial stigma-related concerns among MSMW. Reports of these negative psychological experiences clustered around key themes including high levels of anxiety, psychological distress and depression, alcohol and drug use, suicidal ideation and suicide attempts.

#### Anxiety, psychological distress and depression

In-depth interviews provided evidence of impact on mental health, as all MSMW reported that same-sex sexual behavior remains highly stigmatized in Haiti. As a result, anxiety, psychological distress, and depression are found to be directly linked to widespread harassment and violence.

“I felt like I didn’t have a place in society. They were saying that I had AIDS, that's what made me go get a rope to hang myself. Sometimes it’s shame… Sometimes, I can say, I don’t care if I die, I don’t care, since I’m already dead.” IDIParticipant009.

#### Social isolation, alcohol and other drug use

Furthermore, participants described substance use as a path to avoid any kind of discrimination and mental discomfort in their family or community. Consequently, as described by other studies, when they are drunk and/or under the influence of other drugs, they indulge in unsafe sex^8^.

“I smoke and I drink. I drink more when I have something happening to me like stress, and other things too. I’ll take shots of alcohol to pass the stress. When I drink, I feel like I’m not in the same state, it helps me pass the stress. And, I smoke marijuana. It helps me forget” IDIParticipant010.

#### Suicidal ideation and suicide attempt

While respondents stated that there were few resources for psychological counseling or medical treatment for depression available to them, participants across different backgrounds and demographic characteristics feared the consequences of potential disclosure of their same-sex sexual behavior as well as their HIV status. Thoughts and attempts of suicide as reactions were commonly identified as the only possible response to disclosure, since they believed that they had no alternative.

“Well when I felt like I lost hope for my life is when I learned that I have HIV in my blood, and when I did the test, I learned that, at the time I felt like committing suicide, I said life is over” IDIParticipant011.

### Stigma: coping mechanisms

Little attention has been paid to social barriers and coping mechanisms through the lived experiences and mechanisms of MSMW. Thus, a part of our research focused on identifying stigma coping mechanisms that can be used for successful implementation of HIV prevention and care among MSMW. Analysis of the in-depth interviews identified three main themes for mechanisms: psychological, interpersonal, and structural.

#### Psychological mechanism: self-acceptance

Participants reported that internal and perceived stigma were exacerbated in accordance with perceived values surrounding masculinity and reinforced by culture and religion. However, acceptance of sexual identity and HIV status was a prominent determinant for well-being and strong social support networks. Thus, psychosocial constructs are mandatory to improve their overall health and therefore HIV prevention and care engagement:

“I said, I’m becoming a gay. I reached a place where I said I didn’t want to be gay but I finally became gay. Nothing makes me feel bad. I say life continues. I don’t take drugs. I don’t take alcohol.” IDIParticipant012.

#### Interpersonal mechanisms: social support and hiding sexual orientation

Several participants explained that social support from friends and family help ameliorate sexual and HIV-related stigma.

“I have 2, 3 friends who give me support, I get the same from close family. Sometimes I would feel like I have problems inside of me, after that I realize that I sometimes feel tormented but I resign myself. I sometimes talk to my friends, I tell them I don’t know what to do anymore.” IDIParticipant013.

Homosexual practices are driven underground because cultural constructions of masculinity guide the norms of acceptable behavior. These factors explain why our participants are involved with both male and female sexual partners, and sometimes they appear to adopt a socially acceptable heterosexual lifestyle. Marrying women and fathering children are, for some, a strategy to avoid the negative consequences of public disclosure of homosexuality and can be used to help dispel doubts about masculinity. By having female sexual partners, MSMW fulfill the traditional gender roles and respect the heteronormative and hegemonic model of masculinity. However, in this way, structural factors are interconnected and converge to increase individual risk practices, thereby also increasing both social and other individual drivers of HIV vulnerability.

“If I do things with women it’s to please my mother. Because I’m my mother’s child, I love her. She would like to go to my wedding; I have relationships with women. But I might just have had sex with a woman, but I feel like I should have sex with a man for me to feel satisfied.” IDIParticipant014.

“When I was 18, 19 years old, I had a girlfriend. I had a baby with her. She became pregnant but I got to a point where it wasn’t in me anymore. Deep inside me, I didn’t like women for real. But I had to publicize myself with women when I was a man with man relationships.” IDIParticipant015.

#### Structural mechanism: refuge in the voodoo community

As Christian religion and religiosity are considered to have a connection with stigmatization toward MSMW, establishing social and spiritual networks is conducive to better social well-being. Haitian Voodoo is an ancestral folk religion whose tenets have always been queer-friendly as per their expressions. Therefore, many participants reported finding tolerant attitudes and acceptance in the voodoo community. Participants commonly associated affiliation to the voodoo community with a way to find refuge, and to openly express and celebrate who they were:

“When I was growing up, I was a practicing Protestant, but in regards to my functioning, knowledge, and my relationship with men, I practice voodoo.” IDIParticipant017.

“[Stigmatizing Christian messaging] hurt me a lot, I didn't feel well at all. That's what caused me to leave the church even more and for me to go to voodoo. Currently, I'm in voodoo.” IDIParticipant018.

## Discussion

Previous studies of HIV-infected MSMW have documented the prevalence of stigma and the cross-sectional associations to mental health, HIV risk behaviors, and ART care^34,35^. However, the fuller context of the MSMW experience in resource-limited Caribbean settings such as Haiti is often not pursued. Specifically, the patterns of stigma endured, and sexual development, mental health impact, and coping mechanisms remain thinly studied, undercutting intervention development. Therefore, the motivation of the present work was to find the missing texture in the MSMW experience by pursuing these investigations through a cultural lens, seeking the voices and testimony of these men through qualitative design.

This paper may be the first published qualitative study to explore the development of sexual identity and the patterns of stigma among MSMW living with HIV in Haiti. Our study revealed three main findings: (a) MSMW experienced many forms of stigma under heteronormative and religious pressures: verbal and physical harassment, family and community rejection, violence, and employment discrimination; (b) stigma had mental health consequences: anxiety, psychological distress, depression, social isolation, alcohol and other drug use, suicidal ideation and suicide attempts; and (c) a structure of coping mechanisms and frequent strategies were identified: psychological (self-acceptance), interpersonal (social support and covering sexual orientation), and social structural (refuge in voodoo community).

Our findings reveal the broader context of sexual and HIV-related stigma towards MSMW living with HIV. In order to genuinely capture the main constituents of stigma, we started with an exploration of sexual development and identity formation. As one of the key factors leading to stigma, heteronormativity is a rarely questioned and addressed concept when it comes to interventions for MSMW. The Caribbean cultural constructions of masculinity impose social obligations and restrictions^36^. Thus, any other form of sexual orientation outside the norm is seen as a deviant act. These findings are in line with other studies from the region as well as in other settings^36–38^.

Family plays a central role in Haiti as adult children often continue to live with their parents or pay frequent visits. However, the family as a moral and social resource is very circumscribed for MSMW, especially for those living with HIV. The majority of the participants reported that family and community attitudes are marked by great hostility and violence. These attitudes prevent disclosure of sexual orientation to family and parents, thus stunting self-acceptance of sexual identity during development and exacerbating vulnerability to HIV^39–41^.

Negative attitudes, behaviors, and judgments towards individuals living with HIV interact synergistically with discrimination and marginalization based on sexual orientation for those who are MSM or MSMW, entrenching HIV deeper in communities^42,43^. In Haiti, this “double victim” effect leads to physical, verbal and mental abuse, psychological and physical violence, community and systemic discrimination, and reduced health access to and uptake of HIV services^44,45^.

Evidence of enacted and internalized stigma emerged in several of the interviews, with real mental health consequences. We found that a considerable number of participants had experienced multiple psychosocial issues including early experiences of abuse, social and family rejection, depression, isolation, drug use, suicidal ideation and suicide attempts. Other studies have demonstrated that poor mental health is particularly linked to a high risk of engaging in unsafe sex, acquiring HIV, and failing to initiate or engage in ART if seropositive^46–48^. Thus, a combination prevention approach may be critical for MSMW, in which behavioral interventions and mental health treatment are integrated to provide sustained support of HIV risk reduction. As no single HIV intervention can necessarily change the state of the environment in which MSMW live, the current findings suggest a focus on MSMW’s mental well-being by improving the resources for coping with stigma.

Three types of mechanisms for coping with stigma were described by participants. The first, self-acceptance, brings comfort with sexual orientation and facilitates disclosure to family and parents, which may decrease mental health issues and HIV risk behaviors. These findings were also found in other studies^49,50^. Secondly, as interpersonal mechanisms, social support and living a ‘double life’ by covering sexual orientation were revealed by all the participants as ways through which they cope with stigma. While social support has been proven to be a potential factor that can serve as an intervention target, concealing sexual identity by falsely attempting to portray oneself as heterosexual or bisexual has been shown to negatively impact well-being, self-esteem and mental health^51,52^.

Due to highly conservative Christian religious influences on the society, coping with HIV and sexual stigma is challenging for MSMW. However, Haitian Voodoo represents an ancestral queer-friendly religion and community where they find refuge and acceptance. Our study affirms several previous research findings, however the link between structural coping mechanism and voodoo religion is original to this paper. Policy makers need to be knowledgeable about the culture in order to design, test and adapt tailored HIV interventions for MSMW. Concepts relevant to broader Voodoo beliefs and practices can shape attitudes and compliance with treatment and therefore must be considered, as there remains a strong need for increased mental health and social support services that directly target the needs of MSMW living with HIV in Haiti.

Our study has certain limitations that should be kept in mind. The participants included only MSMW who are enrolled in HIV care. Therefore, we may have missed some key experiences related to psychological effects of sexual and HIV stigma that are not well-represented in populations in care, which may underestimate the effect on mental health; this fact may limit the generalizability of the study findings. However, we tried to improve external validity by selecting participants from different backgrounds and facilities. The study was conducted exclusively in Port-au-Prince, Haiti’s capital city. Thus, we cannot confirm whether the results generalize to other parts of the country. It is possible that conceptualization of homosexuality and stigma differ in other locations and small villages with stronger Voodoo influences.

## Conclusions

While we readily acknowledge that our findings require confirmation with more systematic inquiry in larger samples, our findings indicate that whether it is sexual, HIV-related or both, stigma can have profound effects on MSMW. Future interventions to address stigma in order to improve HIV services uptake and retention need to acknowledge the contextual factors and cultural influences while using multi-level interventions. At the individual level, cognitive-behavioral approaches can be helpful in teaching individuals how to restructure their thinking to reduce self-blame and shame while obtaining social support from their environment and linkage to supportive organizations and social networks. Furthermore, the finding that a number of participants in our study found refuge in the voodoo religion points to an alternative belief system that they found to be supportive and tolerant because it challenges western religious conceptions of homosexuality as pathological and/or immoral. This culturally accepted belief system could be used to foster collaboration between voodoo priests and medical providers to improve HIV services uptake and retention in care (“Supplementary Information”).

## Supplementary Information

Supplementary Information.

## Data Availability

Materials and data are available upon request.

## Code availability

Codes are available upon request.
