# Supplementary material for: Patterns of sexual and HIV-related stigma among men who have sex with men and women living with HIV in Haiti
Source: Sci Rep. 2022 May 7;12:7511. doi: 10.1038/s41598-022-11647-1 (PMC9079062; doi:10.1038/s41598-022-11647-1)
Supplement: Supplementary file 1 — Supplementary Information. [file 41598_2022_11647_MOESM1_ESM.pdf]

MSM Project FIU-Les Centres GHESKIO: In-depth Interview Guide

1. **Explain the purpose of the interviews:** "Before we begin, I would like to thank you for agreeing to this interview with me; I would like to give you information about how this will work. The GHESKIO Centers chose you to be part of a study with many other males like yourself who are living with the virus and that in the past year have had sexual relations with other men. We are asking you and the other men to participate in interviews so that we can learn more about your life history, the circumstances under which you learned that you were HIV+, how it affected you and your relationships with your family, your friends, and sexual partners. We would also like to find out about your access to mental health and HIV care services, your patterns of use of these services, the obstacles you face to maintain adherence to prescribed medicines as well as what facilitates your adherence to treatment. We are interested in learning more about your mental health, your history of depression and your use of alcohol and other drugs. In closing, we will also ask you to share your experience with disclosure of your HIV status to others and with stigma and discrimination.

2. **Explain confidentiality of interview** : "I want to give you the assurance that I will keep everything you tell me secret. This means that no one will know what you said. When the interviews are done we will write a report where the information that we get from all those involved in these interviews will be summarized together. Your name will not appear in this report. Your participation in these interviews will not take away any benefit or any right you have here or with any other organization that gives you support or services. We understand that it may be difficult for you to talk about deeply personal experiences. This is why you can ask me to stop the conversation for a moment or to end it at any time. Do you have any questions?"

3. **Ask permission to record interview**: "To be sure that we do not forget anything of what will be discussed today, we will record all the interviews. When the report has been written and we are finished with the information, we will destroy all recordings. Do you have any questions about that?""

4. **Ask the volunteer to sign consent form**: [if form is unsigned] "Before we begin, I am going to ask you to sign this consent form which includes all that I just explained to you and states that you decided on voluntarily to participate in this interview. Thank you."

<u>Questions for the Interview</u>:

1. **<u>Getting Acquainted</u>**:

   Fill out demographic data form

   [This will give you the opportunity to learn something about the interviewee]

2. **Let's talk about your growing up**

   "Now, would you describe to me your experience growing up up from a very young age to about the age of 11 ? [Give time to respond and probe as needed]

   [Some topics that might be explored: Where he was born/grew up ; the school/schools he attended ; the type of family he grew up in ; his family's socioeconomic status ; his friends ; history of violence/trauma in childhood]

3. **What about your adolescence ?**

   "Describe to me your experiences as you entered puberty and adolescence?" [Give time to respond and probe as needed]

   [Some topics that might be explored:: Sexual attractions and experiences with males and females; sexual identity during this developmental period; individuals he identified with and confided in ; the role that religion played in his infancy and adolescence]

4. **Talk about your life here in Port-au-Prince now.**

   « Would you describe your life here in Port-au-Prince ? » [Give time to respond and probe as needed]

   [Some topics that might be explored : How he came to live in the capital (if not born there) ; current living situation (alone/with other people) ; stability of housing ; type of neighborhood ; employment status ; economic situation ; friends or support system ; experience with exchange of for money or housing]

5. **Let's now talk about your health and how you take care of yourself?**

   "Now, I'd like you to describe the circonstances where you learned about your HIV status and how you have coped over time. » [Give time to respond and probe as needed]

   « Would you describe what type of medicines the doctor has prescribed for your HIV and your experience taking them ? [Give time to respond and probe as needed]

   [Some topics that might be explored: The barrers to adherence (e.g., stigma, distance to clinic, food insecurity, lack of disclosure to family, friends, sexual partners, etc..) ; Facilitators of adherence (e.g., social support from family, friends, partner, support group at clinic/community organization ; reminders, etc..)]

6. **Let's talk about your emotional and psychological health?**

« Describe how you have been feeling emotionally over the past few months ? » [Give time to respond and probe as needed]

[Some topics that might be explored: Mood in general (feelings of depression, anxiety, anger/frustration ; other times in his life where he has felt depressed ; circumstances that might have led to feelings of depression ; coping strategies ; access to treatment and/help sought ; history of suicidal ideations and/or attempts]

7. **Let's talk about alcohol and drugs use in your life :**

« Tell me about the circumstances where you consume alcohol» [Give time to respond and probe as needed]

« Tell me about the circumstances where you use drugs» [Give time to respond and probe as needed]

[Some topics that might be explored for both alcohol and other drug use (AOD): history of use, pattern and frequency of use ; use proximal to sex ; social network for AOD use ; types of AOD, etc.]

8. **Let's talk about your sexual relationships**

« Tell me about your sexual relationships with male partners» [Give time to respond and probe as needed]

[Some topics that might be explored: type of partners (e.g., regular, casual, one partner at a time/multiple partners at a time); serostatus of partners ; use of condoms with different partners ;disclosure of status to partners; reasons for disclosure/non-disclosure, exchange of sex for money/housing/drugs, etc..]

« Tell me about your sexual relationships with female partners» [Give time to respond and probe as needed]

[Some topics that might be explored: type of partners (e.g., regular, casual, one partner at a time/multiple partners at a time); serostatus of partners ; use of condoms with different partners ;disclosure of status to partners; reasons for disclosure/non-disclosure, exchange of sex for money/housing/drugs etc..]

9. **Finally, let's talk about your experiences with stigma, discrimination, and violence**

« Describe for me situations you’ve been in where you’ve felt stigmatized or discriminated against because of your HIV status or your sexual orientation. » [Give time to respond and probe as needed]

[Some topics that might be explored: feelings and reactions in the situations described]

[For each situation, find out how he felt and reacted]

« Describe for me situations you’ve been in where you’ve experienced violence from strangers ? From intimate sexual partners ? [Give time to respond to each question and probe as needed]

[Some topics that might be explored: feelings and reactions in the situations described ; circumstances that triggered the acts of violence]

10. <u>Conclusion of Interview:</u>

Thank participant for taking the time for the interview and for his contributions to the project. Ask if he has any questions or comments he would like to add. Find out if he feels ok about ending the interview at this time.
